# A Strategy for Classification of “Vaginal vs. Cesarean Section” Delivery: Bivariate Empirical Mode Decomposition of Cardiotocographic Recordings

**DOI:** 10.3389/fphys.2019.00246

**Published:** 2019-03-19

**Authors:** Saqib Saleem, Syed Saud Naqvi, Tareq Manzoor, Ahmed Saeed, Naveed ur Rehman, Jawad Mirza

**Affiliations:** ^1^Department of Electrical and Computer Engineering, COMSATS University Islamabad, Sahiwal, Pakistan; ^2^Department of Electrical and Computer Engineering, COMSATS University Islamabad, Islamabad, Pakistan; ^3^Energy Research Center, COMSATS University Islamabad, Islamabad, Pakistan; ^4^School of Computing, Ulster University, Newtownabbey, United Kingdom

**Keywords:** fetal heart rate, uterine contraction, bivariate empirical mode decomposition, intrinsic mode function, vaginal delivery, cesarean section

## Abstract

We propose objective and robust measures for the purpose of classification of “vaginal vs. cesarean section” delivery by investigating temporal dynamics and complex interactions between fetal heart rate (FHR) and maternal uterine contraction (UC) recordings from cardiotocographic (CTG) traces. Multivariate extension of empirical mode decomposition (EMD) yields intrinsic scales embedded in UC-FHR recordings while also retaining inter-channel (UC-FHR) coupling at multiple scales. The mode alignment property of EMD results in the matched signal decomposition, in terms of frequency content, which paves the way for the selection of robust and objective time-frequency features for the problem at hand. Specifically, instantaneous amplitude and instantaneous frequency of multivariate intrinsic mode functions are utilized to construct a class of features which capture nonlinear and nonstationary interactions from UC-FHR recordings. The proposed features are fed to a variety of modern machine learning classifiers (decision tree, support vector machine, AdaBoost) to delineate vaginal and cesarean dynamics. We evaluate the performance of different classifiers on a real world dataset by investigating the following classifying measures: sensitivity, specificity, area under the ROC curve (AUC) and mean squared error (MSE). It is observed that under the application of all proposed 40 features AdaBoost classifier provides the best accuracy of 91.8% sensitivity, 95.5% specificity, 98% AUC, and 5% MSE. To conclude, the utilization of all proposed time-frequency features as input to machine learning classifiers can benefit clinical obstetric practitioners through a robust and automatic approach for the classification of fetus dynamics.

## 1. Introduction

According to the World Health Organization (WHO) (World Health Organization et al., 2015), the high global pregnancy-related mortality ratio of 216 per 100,000 live births is caused by the postpartum hemorrhage, infections and pre-eclampsia (World Health Organization, 2015; Withers et al., 2018). The postpartum hemorrhage, a leading cause of maternal deaths (Say et al., 2014), is mainly quoted because of the excessive blood loss and uterine atony. The substandard care, in terms of imprecise blood loss estimate and delayed involvement of trained obstetricians, is also key to maternal mortality and morbidity (Crowhurst and Plaat, 1999; Rizvi et al., 2004). This underpins a computerized risk score system using continuous monitoring of the fetus for early identification of associated risks during antepartum and intrapartum periods.

Fetal heart rate (FHR) monitoring is the most common procedure assessing the fetal health in the present-day obstetric practice (Devane et al., 2017). For this, various techniques are in practice including fetal stethoscope, intermittent auscultation (Doppler ultrasound) and electronic fetal monitoring (EFM) (Freeman et al., 2012). These techniques have the potential to determine intrauterine hypoxia (Alfirevic et al., 2017), and also make additional assessments leading to the identification of normal and abnormal births (Alfirevic et al., 2017). Though fetal stethoscope is cheap and easy to use for monitoring purposes only, it lacks the recording of FHR and also requires right expertise to interpret. Similarly, intermittent auscultation provides baseline FHR along with the baseline variability, accelerations and decelerations, however, their quantification also remains daunting (Rahman et al., 2012). On the contrary, the EFM, also named cardiotocography (CTG), provides not only the precise monitoring and recording of FHR but also captures maternal uterine contractions (UCs), making CTG a more attractive technique in obstetrics (Warrick et al., 2009).

The current obstetric litigation mostly relies on the visual assessment of the CTG adhering to guidelines provided by the International Federation of Gynecology and Obstetrics (FIGO) (Rooth et al., 1987). However, the subjective interpretation, and high inter- and intra-observer heterogeneity of the CTG has led the current research to investigate and propose novel computerized quantitative and objective measures which might assist obstetricians in their clinical practice. To date, various signal processing algorithms and machine learning paradigms have been utilized to quantify temporal dynamics of CTG tracings. For example, Signorini et al. (2003) proposed a multiparametric scheme based on the linear (autoregressive) and nonlinear (approximate entropy) models for the FHR analysis. Similarly, Ferrario et al. (2006) adopted variants of entropy measures to capture qualitative variations of FHR patterns. Recently, machine learning algorithms based ensemble classifier, trained on features extracted from raw FHR records, has been proposed for the robust detection of intrapartum fetal acidosis (Spilka et al., 2017), and vaginal vs. cesarean delivery (Fergus et al., 2018). Most of these attempts focused solely on FHR dynamics. Whilst, being integral to the autonomous nervous control, FHR variability is sensitive to intrinsic and/or extrinsic stimuli, for example, UCs (Romano et al., 2006).

Along these lines, Warrick et al. (2009) and Warrick et al. (2010) performed a series of studies to quantify the dynamic coupling of UC (as an input) and FHR (as an output). In Warrick et al. (2009), a linear system identification approach was applied to quantify strength and timing of the FHR response to UC in terms of the gain and delay of the estimated impulse response function (IRF). A follow up study (Warrick et al., 2010) employed an integrated approach of system identification along with the FHR baseline and variability features to classify normal and hypoxic fetuses. These studies accounted for only linear components and also assumed UC-FHR dynamics to be stationary. Casati et al. (2014) utilized a phase based signal processing algorithm, namely bivariate phase-rectified signal averaging (BPRSA), to investigate the non-stationary coupling of UC-FHR patterns. Though these methods have performed satisfactorily, their underlying assumption of linearity or stationarity among UC and FHR signal components have resulted in limited accuracy in practical scenarios; thereby motivating the application of modern signal processing approaches accounting for both inherent (nonlinearity and nonstationarity) complexities of UC-FHR dynamics.

Empirical Mode Decomposition (EMD) is a data-adaptive signal decomposition method which has been designed specifically for nonlinear and nonstationary signals (Huang et al., 1998). Recently, multivariate extensions of EMD have emerged (Rilling and Flandrin, 2007; Rehman and Mandic, 2010) which are capable of modeling complex dynamic interactions between multiple input data channels, while still accounting for the nonlinearity and nonstationarity of input data. These attributes have resulted in wide-ranging applications of EMD e.g., from biomedical signal processing (Zahra et al., 2017) to data fusion (Rehman et al., 2015a,b) and signal denoising (Hao et al., 2017). While the EMD based time-frequency features, in combination with support vector machines (SVM) as a classifier, have been employed for the classification of FHR recordings as “normal” or “at risk” (Krupa et al., 2011), to our knowledge, the potential of multivariate extensions of EMD to cater nonlinear and nonstationary complex interactions between UC and FHR signal components for the classification of different fetal states is yet to be explored.

In this study, we propose to use multivariate extensions of EMD to derive robust features that are based on nonlinear and nonstationary neural interactions of FHR and UC signals, in order to classify fetal states leading to “vaginal” vs. “cesarean section” delivery. We hypothesize that complex features associated with natural oscillations of UC and FHR couplings may be utilized to discern vaginal and cesarean temporal dynamics. To test this hypothesis, 552 CTG tracings of an open access CTU-UHB database were examined using a novel data-adaptive approach of bivariate EMD (BEMD) (Rilling and Flandrin, 2007) to derive intrinsic mode functions (IMFs) of UC and FHR recordings. Subsequently, a set of features was extracted from each IMF of UC and FHR series, and tested by adopting machine learning classifiers.

## 2. Materials and Methods

The data analyzed in the present study was taken from a freely available CTU-UHB intrapartum cardiotocography database available at Physionet: http://www.physionet.org/physiobank/database/ctu-uhb-ctgdb/ (Goldberger et al., 2000). A detailed description of this database can be found in Chudáček et al. (2014); herein a brief overview is provided.

### 2.1. Data Collection

The STAN S21 or S31 (Neoventa Medical, Molndal, Sweden), and Avalon FM40 or FM50 (Philips Healthcare, Andover, MA) fetal monitors were used for CTG recordings of FHR (measured in beats/min) and UC (measured in mmHg) waveforms. An ultrasound transducer attached to the abdominal wall was used to record FHR, while a pressure transducer connected to the maternal abdomen was used to record UC; both sampled at 4 Hz. The dataset comprised a total of 552 CTG tracings with singleton, uncomplicated pregnancies; no known intrauterine growth restriction or congenital defects; gestational age > 37 weeks; maternal age > 18 years; 506 vaginal deliveries and 46 cesarean section. Each record was of maximum 90 mins duration preceding the delivery, including stage-I recording of maximum 60 min and stage-II recording of maximum 30 min. The dataset also contains the following clinical and outcome measures: sex, weight, gestational age and presentation of the fetus, type of delivery, lengths of labor (I & II) stages, parity, umbilical artery pH, base excess, base deficit in extracellular fluid, Apgar scores, and partial pressure of CO_2_ (pCO_2_).

### 2.2. Signal Pre-processing

CTG recordings in the clinical set-up are prone to various types of noises and artifacts. For example, the sensor contact loss might generate an abrupt FHR drop followed by its sharp restoration, and also fluctuations in the UC baseline. A sliding median filtering was adopted to correct outliers and missing data, using a sliding window of 10 sec and a threshold of (1 ± 0.33)$\bar{\mu }$, where $\bar{\mu }$ is the median computed over the 10 s window at each time instant (Spilka et al., 2017). Missing data lasting more than 10 s was removed for subsequent analysis.

**Figure 1:**
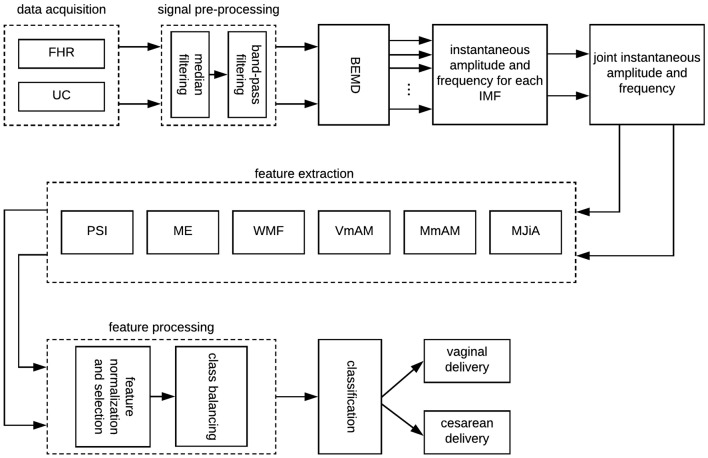
A schematic of the proposed scheme in this study. FHR, fetal heart rate; UC, uterine contraction; BEMD, bivariate empirical mode decomposition; MJiA, mean of joint instantaneous amplitude; MmAM, mean of monotonic change in AM; VmAM, variance of monotonic AM change; WMF, weighted mean frequency; ME, mean energy; PSI, phase synchronization index; IMF, intrinsic mode function.

Traditionally, FHR variability is quantified across arbitrary defined frequency bands. For example, Signorini et al. (2014), Gonçalves et al. (2013), Warrick and Hamilton (2013), and Warrick and Hamilton (2014) subdivided the FHR spectrum across four frequency bands of very low frequency (VLF, <0.03 Hz), low frequency (LF, 0.03–0.15 Hz), movement frequency (MF, 0.15–0.5 Hz), and high frequency (HF, 0.5–1 Hz) (Fergus et al., 2018). On the contrary, Improta et al. (2014) adopted slightly different frequency limits for LF (0.05–0.2 Hz) and HF (0.2–1 Hz) ranges. Generally, VLF is attributed to long lasting nonlinear fluctuations, LF to the neuro-sympathetic fetal control, MF to the fetal movement, while HF is associated to the fetal breathing (Signorini et al., 2003). Keeping the objective of the present study (to quantify the physiology of UC-FHR interactions), we focused on a wide range of frequency (0.004–0.5 Hz) to investigate all possible physiological mechanisms which might be of interest. Consequently, UC and FHR recordings were filtered using a 6th order Butterworth band-pass filter of 0.004–0.5 Hz cut-off frequency. The lower cut-off frequency of this band-pass filter ensures the detrending of baseline. A causal filter was used to avoid phase distortion associated with the filtering process. All data pre-processing and analyses were performed using our in-house built MATLAB (version R2018a; Mathworks) routines. A schematic of the overall proposed scheme in this study is shown in Figure 1.

## 3. Bivariate Empirical Mode Decomposition

We employ EMD based approach to model the nonlinearity and nonstationarity of the input UC and FHR time series data. EMD is a fully data driven approach which decomposes a nonlinear and nonstationary signal into its natural oscillations in the form of distinct amplitude/frequency modulated (AM/FM) components, termed IMFs. EMD has advantage over conventional multiscale approaches of windowed Fourier and wavelet transform, which project input signals onto fixed basis functions (Huang et al., 1998), whereas EMD provides fully data driven basis functions in terms of physically meaningful IMFs and instantaneous frequency estimates.

In our case, bivariate extension of EMD, known as BEMD (Rilling and Flandrin, 2007), has been used to decompose the complex (two-channel) input data *z*(*t*) = *F*(*t*)+*jU*(*t*), consisting of FHR *F*(*t*) and UC *U*(*t*) time series as its real and imaginary components, respectively. BEMD operates by decomposing a bivariate (complex) data into its constituent *rotational* modes in multidimensional spaces thereby preserving inherent correlation(s) present within the two input channels of *z*(*t*). The extension from EMD to BEMD is nontrivial since BEMD requires local mean estimation of input data in multidimensional spaces which is not straight forward. The steps required to compute the BEMD are given in Algorithm 1.

**Algorithm 1:** Bivariate EMD

1: Let $\tilde{z}\left(t\right)$=*z*(*t*),
2: a unit complex number $e^{-j\theta_{k}}$ is used to project the complex signal $\tilde{z}\left(t\right)$ in the direction of θ_*k*_ to obtain *K* signal projections, given by $$p_{\theta_{k}}\left(t\right)=ℜ\left(e^{-j\theta_{k}}\tilde{z}\left(t\right)\right),\;k=1,\ldots ,K$$ where ℜ(.) represents the real part of a complex number, and θ_*k*_ = 2*kπ*/*K*,
3: locate ${\left\{t_{j}^{k}\left(t\right)\right\}}_{k=1}^{K}$ corresponding to maxima points of ${\left\{p_{\theta_{k}}\left(t\right)\right\}}_{k=1}^{K}$,
4: obtain envelope curves ${\left\{e_{\theta_{k}}\left(t\right)\right\}}_{k=1}^{K}$ by using the spline interpolation of maxima points $\left[t_{j}^{k},\tilde{z}\left(t_{j}^{k}\right)\right]$,
5: determine the arithmetic mean *m*(*t*) of all envelope curves, and subtract from the input signal i.e., $d\left(t\right)=\tilde{z}\left(t\right)-m\left(t\right)$. Next, let $\tilde{z}\left(t\right)=d\left(t\right)$ and go to step 2,
6: repeat until *d*(*t*) becomes an IMF.

In the present study, modeling of input data in the form of a complex signal *z*(*t*) enables us to exploit inherent correlation among UC and FHR time series data. In addition, it provides useful insight into nonlinear and nonstationary dynamics of data in terms of its instantaneous amplitude and instantaneous frequency estimates. We employ BEMD to produce a set of *M* complex IMFs, γ_*m*_(*t*), *m* = 1, …, *M*, from an input signal *z*(*t*) as follows:

(1) $$\begin{array}{l} z\left(t\right)=\overset{M}{\underset{m=1}{\sum }}\gamma_{m}\left(t\right)+r\left(t\right) \end{array}$$

where *r*(*t*) denotes the trend in data.

Next, the real and imaginary components of the estimated complex IMFs are separated into real-valued IMFs, corresponding to real and imaginary components of *z*(*t*) i.e., real part of γ_*m*_(*t*) will be the *m*th IMF for FHR ($\gamma_{m}^{\text{F}}\left(t\right)$) while the imaginary part will become the *m*th IMF for UC ($\gamma_{m}^{\text{U}}\left(t\right)$).

Subsequently, the Hilbert transform is applied to each IMF to estimate the instantaneous amplitude and instantaneous frequency. For example, the Hilbert transform representation for the *m*th IMF of FHR time series is given by the following analytical signal,

(2) $$\begin{array}{l} \gamma_{m+}^{\text{F}}\left(t\right)=\gamma_{m}^{\text{F}}\left(t\right)+jH\left(\gamma_{m}^{\text{F}}\left(t\right)\right), \end{array}$$

where H is the Hilbert transform operator. The instantaneous amplitude of $\gamma_{m+}^{\text{F}}\left(t\right)$ can be computed as follows,

(3) $$\begin{array}{l} A_{\gamma_{m}^{\text{F}}}\left(t\right)=\sqrt{{\left(\gamma_{m}^{\text{F}}\left(t\right)\right)}^{2}+{\left(H\left(\gamma_{m}^{\text{F}}\left(t\right)\right)\right)}^{2}}. \end{array}$$

The instantaneous phase of $\gamma_{m+}^{\text{F}}\left(t\right)$ can be defined as,

(4) $$\begin{array}{l} \phi_{\gamma_{m}^{\text{F}}}\left(t\right)=\arctan\left[\frac{H\left(\gamma_{m}^{\text{F}}\left(t\right)\right)}{\gamma_{m}^{\text{F}}\left(t\right)}\right]. \end{array}$$

The instantaneous frequency is defined as the rate of change of the instantaneous phase of $\gamma_{m+}^{\text{F}}\left(t\right)$,

(5) $$\begin{array}{l} f_{\gamma_{m}^{\text{F}}}\left(t\right)=\frac{d}{dt}\left[\phi_{\gamma_{m}^{\text{F}}}\left(t\right)\right]. \end{array}$$

The same procedure is followed to obtain the instantaneous amplitude $A_{\gamma_{m}^{\text{U}}}\left(t\right)$, phase $\phi_{\gamma_{m}^{\text{U}}}\left(t\right)$ and frequency $f_{\gamma_{m}^{\text{U}}}\left(t\right)$ for the UC signal.

Finally, the joint instantaneous amplitude $A_{\gamma_{m}}^{\text{multi}}$, and the joint instantaneous frequency estimates $f_{\gamma_{m}}^{\text{multi}}$ belonging to *m*th IMFs of FHR and UC are given by Lilly and Olhede (2012), Ahrabian et al. (2015), and Bhattacharyya and Pachori (2017),

(6) $$\begin{array}{l} A_{\gamma_{m}}^{\text{multi}}\left(t\right)=\sqrt{{\left[A_{\gamma_{m}^{\text{F}}}\left(t\right)\right]}^{2}+{\left[A_{\gamma_{m}^{\text{U}}}\left(t\right)\right]}^{2}} \end{array}$$

(7) $$\begin{array}{l} f_{\gamma_{m}}^{\text{multi}}\left(t\right)=\frac{{\left[A_{\gamma_{m}^{\text{F}}}\left(t\right)\right]}^{2}f_{\gamma_{m}^{\text{F}}}\left(t\right)+{\left[A_{\gamma_{m}^{\text{U}}}\left(t\right)\right]}^{2}f_{\gamma_{m}^{\text{U}}}\left(t\right)}{{\left[A_{\gamma_{m}^{\text{F}}}\left(t\right)\right]}^{2}+{\left[A_{\gamma_{m}^{\text{U}}}\left(t\right)\right]}^{2}} \end{array}$$

## 4. Feature Extraction

The present study utilized a set of following features: mean of joint instantaneous amplitude, mean of monotonic change in amplitude modulation (AM), variance of monotonic AM change, weighted mean frequency, mean energy and phase synchronization index. These features were estimated for each IMF of both FHR and UC signals. A brief description of these features is provided next.

### 4.1. Mean of Joint Instantaneous Amplitude

Mean of joint instantaneous amplitude (MJiA) is calculated by Bhattacharyya and Pachori (2017),

(8) $$\begin{array}{l} \mu =\frac{1}{T}\int_{T}A_{\gamma_{m}}^{\text{multi}}\left(t\right)dt, \end{array}$$

where $A_{\gamma_{m}}^{\text{multi}}\left(t\right)$ represents the joint instantaneous amplitude, defined in Equation (6), and *T* represents the number of data samples of FHR and UC signals.

### 4.2. Mean of Monotonic AM Change

Mean value of monotonic AM change (MmAM) is given by Bhattacharyya and Pachori (2017) and Kawahara et al. (1999),

(9) $$\upsilon =\frac{1}{T}\int_{T}\frac{dA_{\gamma_{m}}^{\text{multi}}(t)}{dt}dt,$$

where $A_{\gamma_{m}}^{\text{multi}}$ represents the joint instantaneous amplitude, defined in Equation (6).

### 4.3. Variance of Monotonic AM Change

Variance of monotonic AM change (VmAM) is given by Bhattacharyya and Pachori (2017) and Kawahara et al. (1999),

(10) $$\sigma =\frac{1}{T}\int_{T}{\left(\frac{dA_{\gamma_{m}}^{\text{multi}}(t)}{dt}-\upsilon \right)}^{2}dt,$$

where $A_{\gamma_{m}}^{\text{multi}}$ represents the joint instantaneous amplitude, defined in Equation (6), and υ is calculated using Equation (9).

### 4.4. Weighted Mean Frequency

The weighted mean frequency (WMF) for the *m*th IMF of the FHR signal is defined as (Oweis and Abdulhay, 2011; Zahra et al., 2017),

(11) $${f^{\prime }}_{\gamma_{m}^{\text{F}}}=\frac{\sum_{t}A_{\gamma_{m}^{\text{F}}}(t)f_{\gamma_{m}^{\text{F}}}^{2}(t)}{\sum_{t}A_{\gamma_{m}^{\text{F}}}(t)f_{\gamma_{m}^{\text{F}}}(t)},$$

where $A_{\gamma_{m}^{\text{F}}}\left(t\right)$ and $f_{\gamma_{m}^{\text{F}}}\left(t\right)$ are, respectively, instantaneous amplitude and instantaneous frequency, defined in Equations (3) and (5), respectively. Similarly, the WMF for the *m*th IMF of the UC signal can be determined.

### 4.5. Mean Energy

Mean energy (ME) contained by the *m*th IMF of the FHR signal is defined by Biju et al. (2017),

(12) $$\begin{array}{l} E_{\gamma_{m}^{\text{F}}}=log\left(\frac{1}{T}\underset{t}{\sum }|\gamma_{m}^{\text{F}}{\left(t\right)}^{2}|\right), \end{array}$$

Similar approach can be followed to determine ME of the *m*th IMF for the UC signal (i.e., $E_{\gamma_{m}^{\text{U}}}$).

### 4.6. Phase Synchronization Index

Phase synchronization index (PSI) between instantaneous phases of *m*th IMFs for FHR ($\phi_{\gamma_{m}^{\text{F}}}\left(t\right)$) and UC ($\phi_{\gamma_{m}^{\text{U}}}\left(t\right)$) is calculated as (Saleem et al., 2016, 2018)

(13) $$\varphi_{m}=\frac{1}{T}\left({\left[\sum_{t}\text{cos}(\Delta \phi_{\gamma_{m}}(t))\right]}^{2}+{\left[\sum_{t}\text{sin}(\Delta \phi_{\gamma_{m}}(t))\right]}^{2}\right)$$

where $\Delta \phi_{\gamma_{m}}\left(t\right)=\phi_{\gamma_{m}^{\text{F}}}\left(t\right)-\phi_{\gamma_{m}^{\text{U}}}\left(t\right)$. *T* represents the number of data samples of FHR and UC signals, and $\phi_{\gamma_{m}^{\text{F}}}\left(t\right)$ and $\phi_{\gamma_{m}^{\text{U}}}\left(t\right)$ are estimated using Equation (4). The value of PSI index φ_*m*_ ranges from 0 to 1 i.e., 0 corresponds to the absence of synchronization, and 1 corresponds to the perfect synchronization.

## 5. Features Processing

### 5.1. Feature Normalization and Selection

The extracted features (defined in section 4) were found having a divergent range of values which might affect the performance of classifiers. Keeping this, a standard mean normalization feature scaling scheme is applied to raw features, and is given by,

(14) $$\begin{array}{l} {𝔉}^{\text{norm}}=\frac{𝔉-mean\left(𝔉\right)}{max\left(𝔉\right)-min\left(𝔉\right)}, \end{array}$$

where 𝔉^norm^ represents the normalized feature, and *mean*(.), *max*(.), and *min*(.) are, respectively, average, maximum and minimum values of the raw feature 𝔉.

This study considered SVM based recursive feature elimination (RFE) (Guyon et al., 2002) strategy as a feature selection criteria to determine a sub-set of highly discriminating features from the entire feature set. RFE is a recursive procedure in which a ranking criterion for feature sub-set is computed from all features based on learned weights from a classifier (i.e., linear SVM in this work) at each iteration, and subsequently a feature with the smallest criterion is removed. Algorithmic details of RFE are described in the Supplementary Material. The optimal number of features were decided based on the performance of the classification model i.e., sensitivity, specificity, area under the ROC curve (AUC) and mean squared error (MSE).

Belsley collinearity diagnostics (Belsley, 1991) procedure was employed to test the collinearity of extracted features. Briefly, this approach detects the sources of collinearity and provides a measure of their strength by exploiting the condition indices. Variance decomposition proportions are adopted to detect interdependent variates, and to estimate the level of degradation in the regression due to dependencies.

### 5.2. Class Balancing

The imbalance distribution of dataset (506 vaginal deliveries as compared to 46 cesarean cases) might cause the classifier to over-fit to the majority (vaginal) class, and the probability to predict the normal vaginal delivery on most of the unseen test data will be high. To evade this biasing, we employed a standard class imbalance handler, namely synthetic minority over-sampling (SMOTE) (Chawla et al., 2002), in our classification framework. SMOTE (Chawla et al., 2002) works by oversampling the minority class by generating synthetic examples in the feature space based on the chosen *k* nearest neighbors from the minority class (see Supplementary Material for more details).

In the present study, we evaluated the classification performances under two conditions: (1) without class balancing (baseline condition), and (2) with class balancing. For class balancing, the training dataset is over- or under-sampled using the SMOTE method i.e., the minority (cesarean) class is over-sampled by 400%, while the majority (vaginal) class is under-sampled by 100%. The test dataset does not undergo any transformation. The train and test splitting, and the class balancing is repeated for every run and the performance of classifiers is averaged over 30 simulations. For baseline evaluation, classifiers were trained on original features (without class balancing), and also averaged over 30 simulations.

### 5.3. Classification Methods

The present study considered three different classifiers: (i) decision trees, (ii) SVM, and (iii) ensemble classifier: AdaBoost. The overall aim is to determine a classifier with the most appropriate decision boundary resulting in the maximum separability of classes. Recent studies have suggested the application of decision trees to evaluate the fetal state from cardiotocogram signals (Yilmaz and Kilikçier, 2013; Kamath and Kamat, 2018). Thereby, we also adopted this well-known methodology (Breiman, 2017) in the current study, which splits the data space into sub-spaces based on input features. Hierarchical tree structures recursively partition the data space into disjoint sets with linear and nonlinear boundaries. We utilized Gini's diversity index (Breiman et al., 2005) as the split criteria owing to its wide usage. The implementation parameters were chosen appropriately to ensure coarse distinctions between classes.

SVM has been widely adopted in classification problems associated with biomedical data (Georgoulas et al., 2006b; Krupa et al., 2011; Moslem et al., 2011; Ocak, 2013). SVM works by identifying the hyperplane that maximizes separation between classes. The most appropriate hyperplane in feature space maximizes margins between the hyperplane and nearest data points. The important factors concerning SVM tuning include a kernel function, box constraints (which control trade-off between margin-violating observations and the training time) and the kernel scale (which scales predictors before computing the kernel function). A Gaussian kernel function is considered in this study due to non-linearly separable nature of data. Optimal values of box constraints and the kernel scale are sought which maximize the validation accuracy.

Ensemble classifiers have also shown promising performance in similar studies (Tomas et al., 2013; Peterek et al., 2014). Therefore, we also consider the AdaBoost classifier (Schapire, 1999) in this study as a representation of ensemble classifiers. The basic principle of boosting is to significantly reduce the error of any weak learner (which is slightly better than random guessing) by combining predictions of many such classifiers. The algorithm trains a weak learner using different distributions of the training dataset and subsequently combines all classifiers produced by the weak learner into a compound classifier. The number of weak learners is set to 20 as a trade-off between the accuracy and the training time. The learning rate is set as to make the number of learning cycles moderate.

### 5.4. Validation Schemes and Performance Measures

Hold out validation is employed for splitting the data using a 80/20 split i.e., 80% of data is held out for the classifier training, and the remaining 20% data is reserved for the classifier testing. Since training and test datasets are randomly sampled from the entire dataset, learning and prediction steps are averaged over 30 epochs using unique seeds. The over-sampling is repeated on the training data in each step. The performance metrics for each method are averaged over 30 runs.

The performance of classifiers is evaluated in terms of sensitivity, specificity, AUC and MSE. Sensitivity is defined as the rate of correct prediction of the cesarean delivery, whereas specificity is considered as a measure of correct prediction rate of the vaginal delivery. AUC (Fawcett, 2006) represents a trade off between sensitivity and specificity, and is recommended as a classification performance tool when datasets are imbalanced. Larger the sensitivity, specificity and AUC values are, the better the classification performance is. Finally, the MSE is considered to quantify differences between predicted and actual class labels. Smaller values of the MSE indicate better performance of classifiers.

## 6. Results

### 6.1. Bivariate Empirical Mode Decomposition

**Figure 2:**
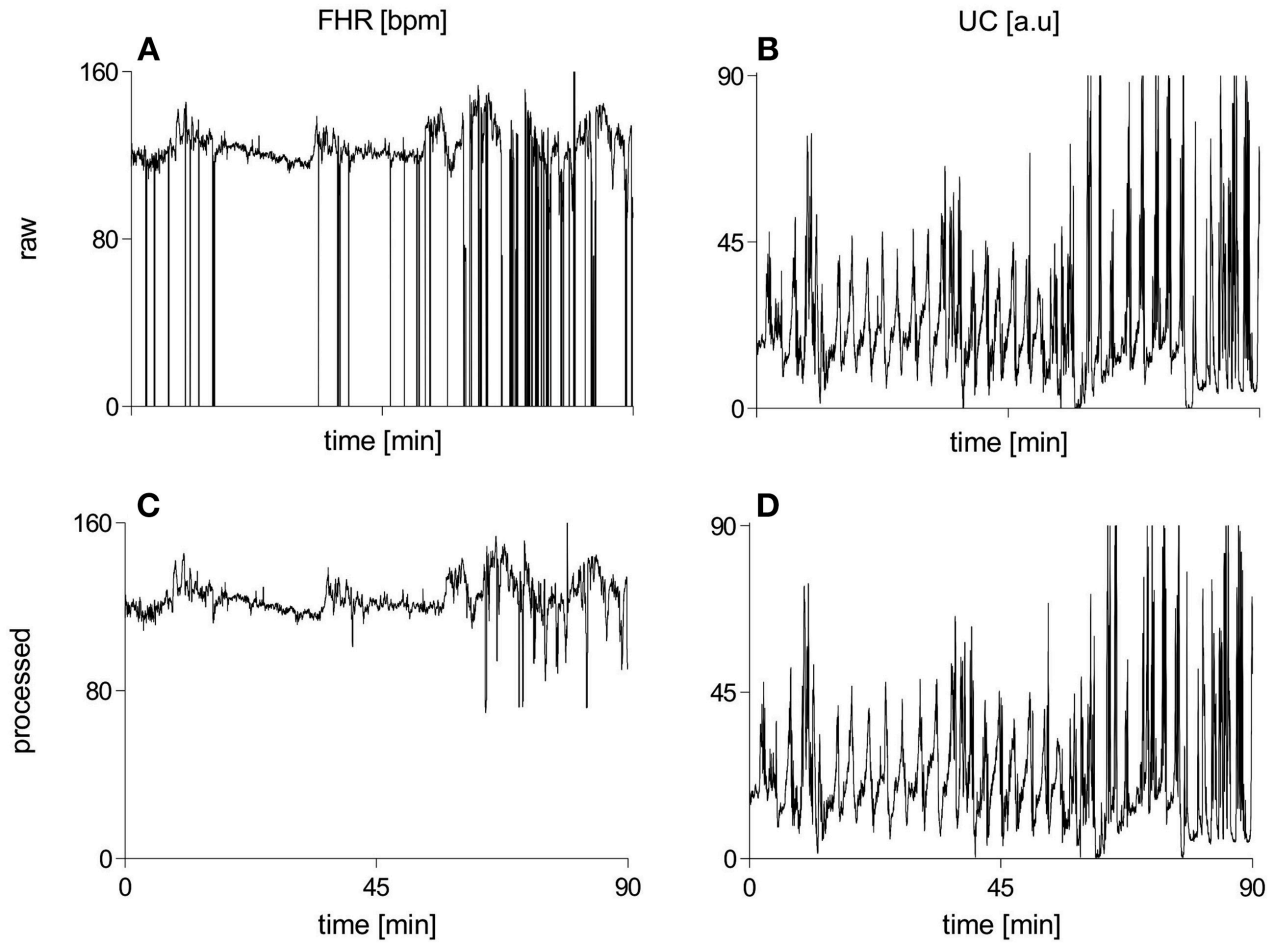
Raw and processed waveforms of FHR [bpm] and UC [a.u] recordings for one representative subject of the vaginal group. **(A)** raw FHR, **(B)** raw UC, **(C)** processed FHR, **(D)** processed UC. FHR, fetal heart rate; bpm, beats per minute; UC, uterine contraction; a.u, arbitrary units.

**Figure 3:**
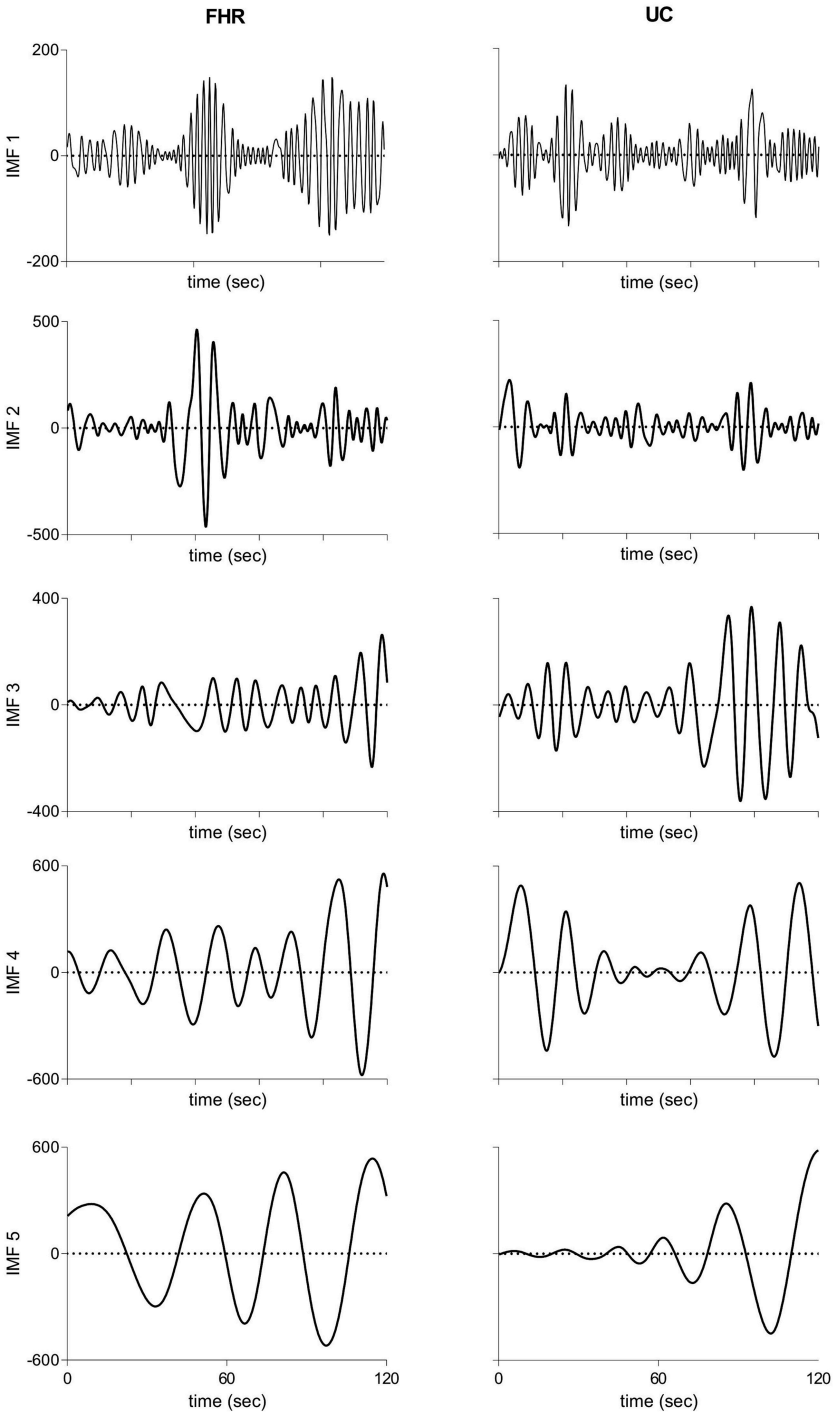
Intrinsic mode functions derived from bivariate empirical mode decomposition of initial 2 mins recordings of FHR **(Left panels)** and UC **(Right panels)** recordings shown in Figure 2. FHR, fetal heart rate; UC, uterine contraction; IMF, intrinsic mode function.

Figure 2 illustrates raw and processed FHR and UC recordings for one representative subject of vaginal cohort. BEMD decomposition, consisting of *M* = 5 bivariate (2-channel) IMFs, of initial 2 mins recordings of FHR (left panels) and UC (right panels) signals (shown in Figure 2) is shown in Figure 3. As expected, it was found that lower-indexed IMFs (i.e., IMF 1) carried higher frequency content while higher-indexed IMFs (i.e., IMF 5) contained lower frequency information. Another important trend that can be observed in the BEMD decomposition is the alignment/matching of similar frequency contents along the same-indexed IMFs from both FHR and UC recordings. This “mode alignment” across multiple IMFs is a characteristic of multivariate extensions of EMD (Rehman et al., 2009) and, in our case, was a vital pre-requisite to obtain robust time-frequency based features for the improved classification. Note that the channel-wise implementation of (single-variate) EMD on our dataset would have yielded mismatched scales across multiple IMFs leading to physically meaningless estimates of chosen features in equations 8–13. Therefore, the application of multivariate extension of EMD to preserve the “matched” scales in FHR and UC recordings is one of the highlights of our work.

Given that we had a large number of subjects in the current study, it was not possible to create a multivariate EMD with that many number of channels due to inherent limitation of multivariate extensions of EMD on total number of input data channels. Therefore, we had applied BEMD separately for each subject, which resulted in matched IMFs across multiple subjects as well and that had been validated empirically by plotting the Fourier spectrum of IMFs obtained from multiple subjects (see Figure S3). The matching of scales across multiple subjects, despite those scales being obtained from multiple instances of BEMD, could be attributed to the fact that natural oscillations of FHR and UC signals were expected to be similar within different subjects.

We tested various selections of M (i.e., number of IMFs), and observed that relevant important information needed for good classification accuracy is present in the first five IMFs. For example, AUC value for the balanced dataset was found to be maximal for *M* = 5 (see Figure S4).

### 6.2. Group-Averaged Features

**Figure 4:**
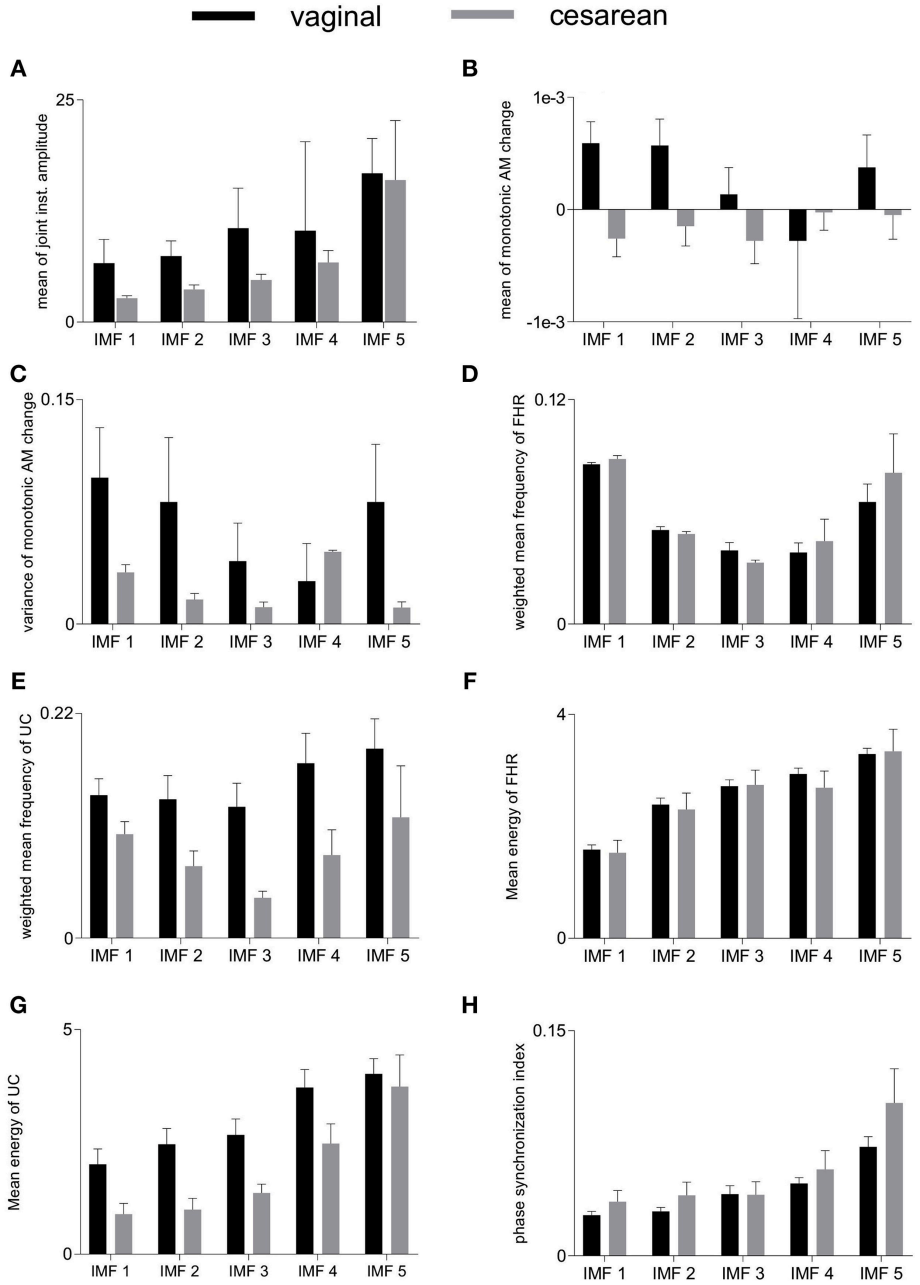
Averaged values of individual features for vaginal and cesarean groups. Error bars represent standard errors. FHR, fetal heart rate; UC, uterine contraction; IMF, intrinsic mode function; AM, amplitude modulation. **(A)** mean of joint instantaneous amplitude, **(B)** mean of monotonic AM change, **(C)** variance of monotonic AM change, **(D)** weighted mean frequency of FHR, **(E)** weighted mean frequency of UC, **(F)** mean energy of FHR, **(G)** mean energy of UC, **(H)** phase synchronization index.

Group-averaged values of individual features evaluated in this study, for each IMF, are given in Figure 4 and also in Table 1. Large values of MJiA were associated with the higher-indexed IMFs (Figure 4A). Vaginal cohort demonstrated greater heterogeneity represented by the large variances associated with all IMFs. For MmAM across the vaginal group, all IMFs were found with positive mean values except the 4th IMF which had negative mean. Whereas, all IMFs for the cesarean group demonstrated negative mean values (Figure 4B). Majority of the MmAM IMFs for the vaginal group were found with larger variances with the 1st IMF having largest and the 4th IMF having smallest variance. As compared to the vaginal group, all MmAM IMFs of the cesarean group demonstrated reduced variance except the 4th IMF which had larger variance than its corresponding IMF for the vaginal group (Figure 4C).

**Table 1:** Group-averages (means ± standard errors) of feature values for vaginal and cesarean groups.

**Features**	**IMF 1**		**IMF 2**		**IMF 3**		**IMF 4**		**IMF 5**	
	**Vaginal**	**Cesarean**	**Vaginal**	**Cesarean**	**Vaginal**	**Cesarean**	**Vaginal**	**Cesarean**	**Vaginal**	**Cesarean**
MJiA (n.a.)	6.6 ± 2.6	2.7 ± 0.3	7.4 ± 1.7	3.7 ± 0.5	10.5 ± 4.5	4.7 ± 0.6	10.3 ± 0.1	6.7 ± 1.3	16.7 ± 3.9	15.9 ± 6.7
MmAM (n.a.)	0.0006 ± 0.0001	−0.0002 ± 0.0001	0.0005 ± 0.0003	−0.0002 ± 0.0001	0.0002 ± 0.0002	−0.0003 ± 0.0002	−0.0003 ± 0.0007	−0.00003 ± 0.0002	0.0004 ± 0.0003	−0.0005 ± 0.0002
VmAM (n.a.)	0.09 ± 0.03	0.04 ± 0.005	0.08 ± 0.04	0.02 ± 0.004	0.04 ± 0.03	0.02 ± 0.003	0.03 ± 0.02	0.05 ± 0.0009	0.08 ± 0.04	0.02 ± 0.003
WMF_FHR_ (rad/se)	0.09 ± 0.0009	0.09 ± 0.001	0.05 ± 0.001	0.05 ± 0.001	0.04 ± 0.004	0.03 ± 0.001	0.04 ± 0.005	0.05 ± 0.02	0.07 ± 0.01	0.08 ± 0.02
WMF_UC_ (rad/se)	0.2 ± 0.01	0.1 ± 0.01	0.2 ± 0.02	0.07 ± 0.01	0.2 ± 0.02	0.04 ± 0.006	0.2 ± 0.02	0.08 ± 0.02	0.2 ± 0.02	0.1 ± 0.05
ME_FHR_ (beats^2^/sec^3^)	1.6 ± 0.08	1.5 ± 0.3	2.4 ± 0.2	2.3 ± 0.3	2.7 ± 0.2	2.7 ± 0.3	2.9 ± 0.1	2.7 ± 0.3	3.3 ± 0.1	3.4 ± 0.4
ME_UC_ (mmHg^2^/sec)	2 ± 0.4	0.9±.3	2.5 ± 0.4	.99±.3	2.7 ± 0.4	1.4 ± 0.2	3.7 ± 0.4	2.5 ± 0.4	4.1 ± 0.4	3.7 ± 0.7
PSI (a.u.)	0.03 ± 0.002	0.04 ± 0.007	0.03 ± 0.002	0.04 ± 0.009	0.04 ± 0.006	0.04 ± 0.009	0.05 ± 0.004	0.06 ± 0.02	0.07 ± 0.007	0.1 ± 0.03

*a.u., arbitrary units; IMF, intrinsic mode function; MJiA, mean of joint instantaneous amplitude; MmAM, mean of monotonic AM change; VmAM, variance of AM change; WMF, weighted mean frequency; ME, mean energy; PSI, phase synchronization index; FHR, fetal heart rate; UC, uterine contraction; n.a., not applicable*.

The highest values of WMF for the FHR recording were observed for the 1st IMF and the lowest value was found for the 3rd IMF for both vaginal and cesarean cohorts (Figure 4D). The WMF variance increased from smaller- to higher-indexed IMFs i.e., the lowest variance occurred for the 1st IMF and the highest variance occurred for the 5th IMF of the FHR series. For the UC series, the highest WMF value was found for the 5th IMF for both vaginal and cesarean groups while the lowest value was found for the 3rd IMF (Figure 4E). Interestingly, all WMF IMFs demonstrated smaller values for the cesarean group, however, this reduction was not found to be statistically significant. Similar to the FHR series, larger variance was also found across the 5th IMF for the UC recording. ME values increased across the ascending order of IMFs for both FHR and UC as well as across both vaginal and cesarean groups (Figures 4F,G). Higher variances were found for all ME IMFs of the cesarean group as compared to the vaginal group. For the UC signal, all IMFs of the cesarean group were found having smaller ME values as compared to the vaginal group. Overall smaller PSI values (< 0.1) were found between FHR and UC with highest values across the 5th IMF and lowest values across the 1st IMF for both vaginal and cesarean groups (Figure 4H). Cesarean group demonstrated higher PSI values as compared to the vaginal group for all IMFs except the 3rd IMF where values remained unaltered.

We compared the group-averaged values of all features for vaginal vs. cesarean differentiation for each IMF. However, no significant differences were observed across any comparison demonstrating that none of these features may solely differentiate between vaginal and cesarean delivery dynamics.

### 6.3. Feature Selection

Belsley collinearity test showed that only five features exhibited dependencies which were of minor strengths. Keeping this, the present work did not perform any feature transformation to remove mutual dependencies. Of note, machine learning classifiers employed in the present work are not influenced by the collinearity because they include parameter tuning (i.e., regularization) which induce implicit feature selection. Moreover, cross-validation also verify the robustness of the employed classifiers even in the presence of collinearity.

**Figure 5:**
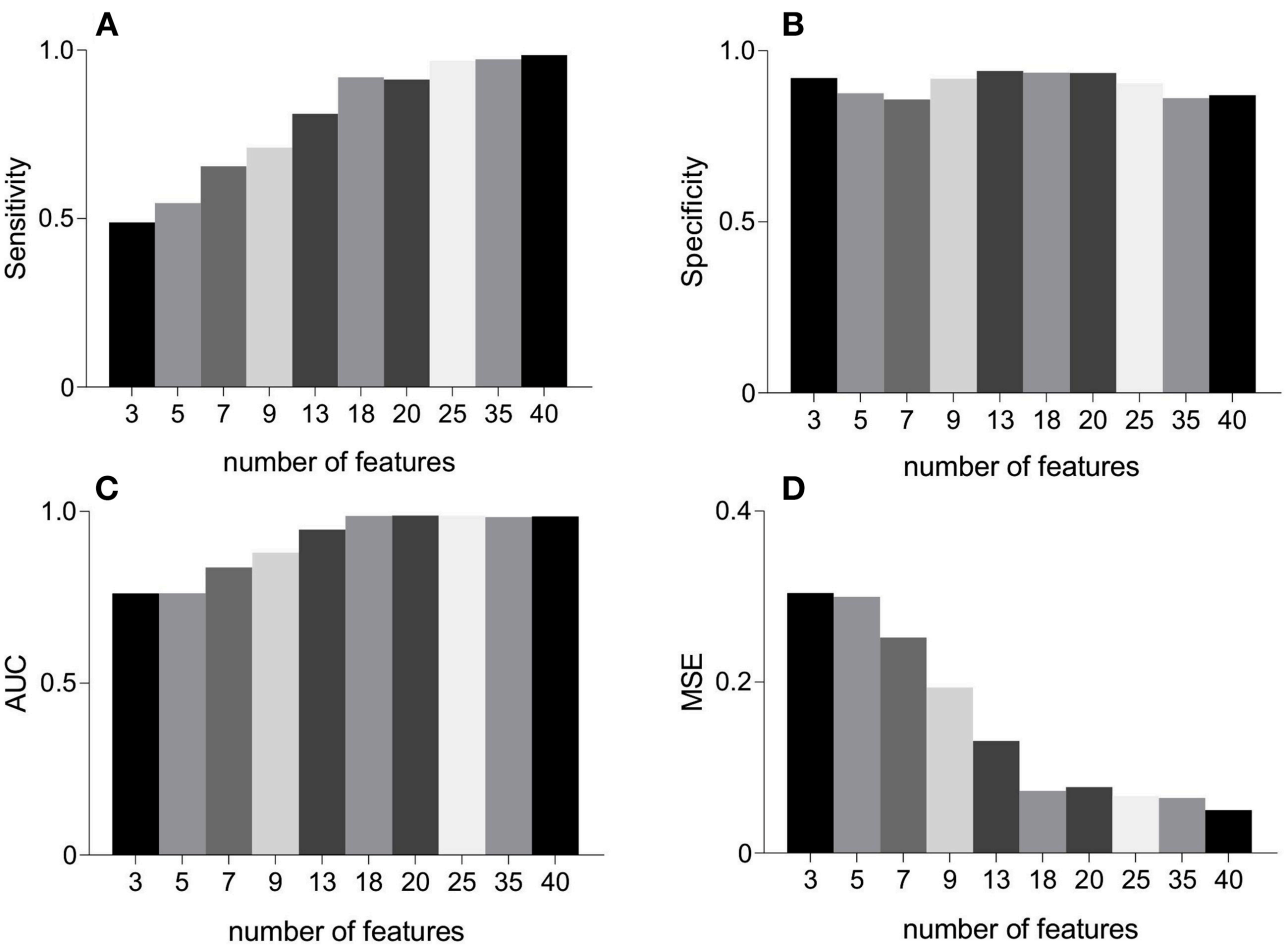
An illustration of performance measures for the RFE feature selection scheme. Panel **(A)** represents sensitivity, **(B)** represents specificity, **(C)** represents AUC, and **(D)** represents MSE. AUC, area under the ROC curve; MSE, mean squared error; RFE, recursive feature elimination.

Figure 5 represents the performance measures for the RFE feature selection strategy as a function of number of features. The highest values for sensitivity and AUC, and lowest MSE were found under the application of all 40 features. Whereas, highest specificity was found for 20 features. These observations signify that discriminating properties of all features are best exploited when employed in a combination. Therefore, we do not consider feature selection in the subsequent classification framework. Combinations of features as a function of number of features (as that of Figure 5) are given in Table 2. For example, AUC of 0.76 was found for combination of 3 features including MmAM of IMF 1, ME of IMF 4, and PSI of IMF 5.

**Table 2:** Combinations of features corresponding to features' selection as of Figure 5.

**Number of features**	**Features**					
	**IMF1**	**IMF2**	**IMF3**	**IMF4**	**IMF5**	**AUC**
3	MmAM			ME of UC,	PSI	0.76
5	MmAM	MJiA		ME of UC, WMF of UC	PSI	0.77
7	MmAM	MJiA	MmAM, ME of FHR	ME of UC, WMF of UC	PSI	0.83
9	MmAM, VmAM	MJiA	MmAM, ME of FHR	ME of UC, WMF of UC	PSI, VmAM	0.88
13	MmAM, VmAM	MJiA, MmAM	MmAM, ME of FHR, ME of UC	ME of UC, WMF of UC, WMF of FHR	PSI, VmAM, MmAM	0.94
18	MJiA, MmAM, VmAM	MJiA, MmAM, ME of FHR	MmAM, ME of FHR, ME of UC, PSI	ME of UC, WMF of UC, WMF of FHR, MJiA	PSI, VmAM, MmAM, ME of UC	0.987
20	MJiA, MmAM, VmAM, ME of UC	MJiA, MmAM, ME of FHR, PSI	MmAM, ME of FHR, ME of UC, PSI	ME of UC, WMF of UC, WMF of FHR, MJiA	PSI, VmAM, MmAM, ME of UC	0.998
25	MJiA, MmAM, VmAM, ME of UC, PSI	MJiA, MmAM, ME of FHR, PSI, ME of UC	MmAM, ME of FHR, ME of UC, PSI, WMF of FHR	ME of UC, WMF of UC, WMF of FHR, MJiA, VmAM	PSI, VmAM, MmAM, ME of UC, ME of FHR	0.988
35	MJiA, MmAM, VmAM, ME of UC, PSI, WMF of FHR, ME of FHR,	MJiA, MmAM, ME of FHR, PSI, ME of UC, WMF of UC, WMF of FHR	MmAM, ME of FHR, ME of UC, PSI, WMF of FHR, WMF of UC, MJiA	ME of UC, WMF of UC, WMF of FHR, MJiA, VmAM, ME of FHR, PSI	PSI, VmAM, MmAM, ME of UC, ME of FHR, MJiA, WMF of FHR	0.983
40	MJiA, MmAM, VmAM, ME of UC, PSI, WMF of FHR, ME of FHR, WMF of UC	MJiA, MmAM, ME of FHR, PSI, ME of UC, WMF of UC, WMF of FHR, VmAM	MmAM, ME of FHR, ME of UC, PSI, WMF of FHR, WMF of UC, MJiA, VmAM	ME of UC, WMF of UC, WMF of FHR, MJiA, ME of FHR, PSI, MmAM, VmAM	PSI, VmAM, MmAM, ME of UC, ME of FHR, MJiA, WMF of FHR, WMF of UC	0.985

*AUC, area under the ROC curve; IMF, intrinsic mode function; MJiA, mean of joint instantaneous amplitude; MmAM, mean of monotonic AM change; VmAM, variance of AM change; WMF, weighted mean frequency; ME, mean energy; PSI, phase synchronization index; FHR, fetal heart rate; UC, uterine contraction*.

### 6.4. Classification Without Class Balancing

**Figure 6:**
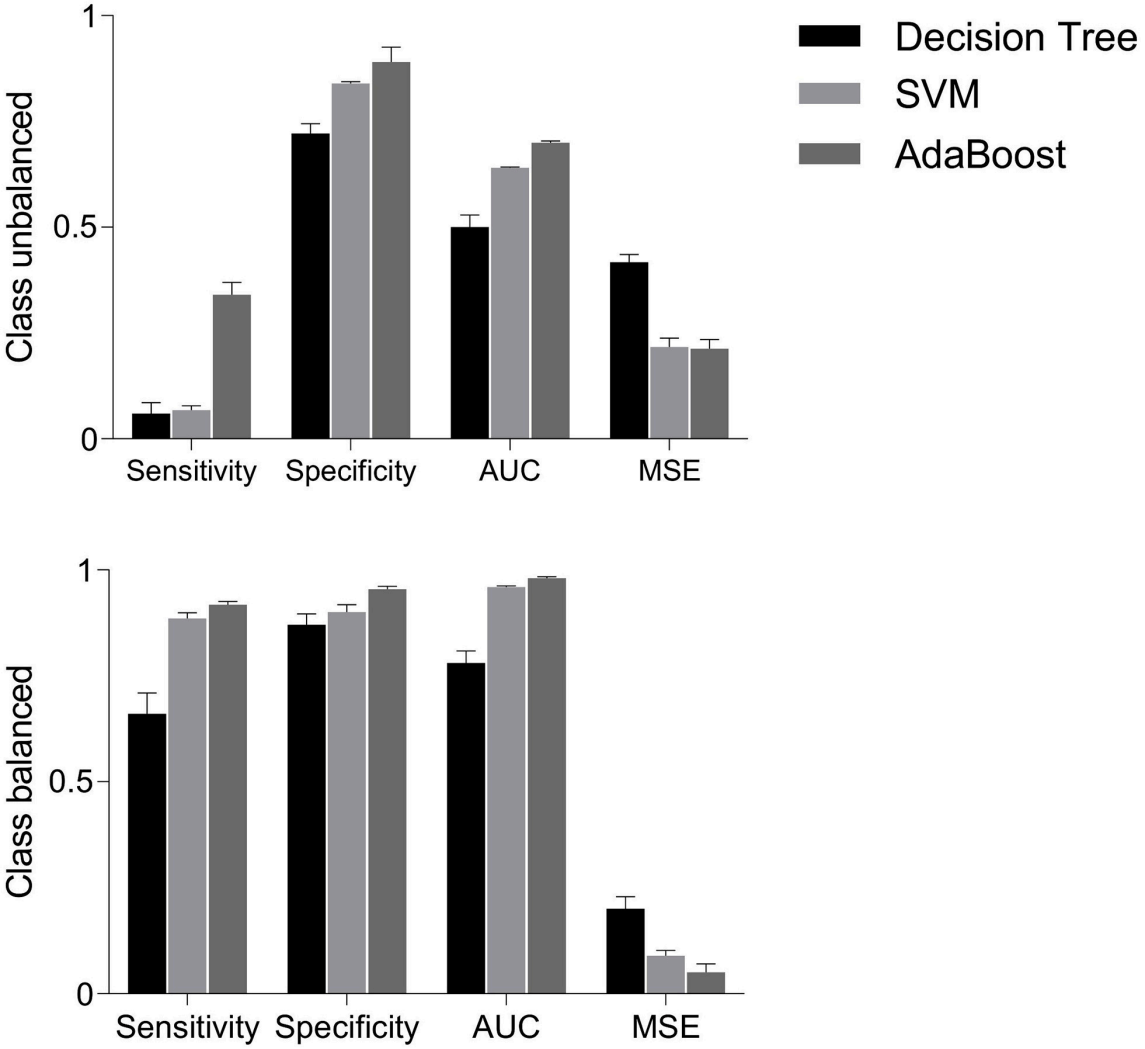
Performance measures i.e., sensitivity, specificity, AUC and MSE, using decision tree, SVM and AdaBoost classifiers for balanced and unbalanced classes. AUC, area under the ROC curve; MSE, mean squared error; SVM, support vector machine.

Figure 6 (upper panels) shows that sensitivities were quite low i.e., 6, 7, and 4%, respectively, for decision tree, SVM and AdaBoost classifiers. However, specificities were relatively higher i.e., 72, 84, and 89% for decision tree, SVM and AdaBoost, respectively. The mis-classification rate depicted in terms of MSE were considerably high i.e., the highest MSE value of 41% was found using decision tree, and the lowest value of 22% was found using AdaBoost. Highest AUC of 70% was observed for AdaBoost while a lowest AUC value of 51% was associated with the decision tree classifier. These results are expected because classifiers are destined to overfit to the vaginal class due to imbalanced data.

### 6.5. Classification With Class Balancing

The classification performance on balanced data is shown in Figure 6 (bottom panels). Sensitivity increased to 66% with decision tree, 88.5% with SVM and 91.8% with AdaBoost; suggesting that the classification of cesarean deliveries can be best performed using an AdaBoost classifier. SVM and AdaBoost maintained highest specificities (90% and 95.5%, respectively) while decision tree demonstrated lowest specificity of 87%; suggesting that the correct prediction of vaginal deliveries can be best performed by an AdaBoost classifier. The AdaBoost classifier outperformed other classifiers in terms of AUC (98%) and MSE (5%) also. To sum, AdaBoost performed the best classification as indicated by higher metrics and also in terms of the confidence on these measures indicated by the reduced standard errors.

### 6.6. Classification Performance Comparison of BEMD vs. EMD

The present study also compared the classification performance of BEMD-based features with that to EMD-based features. For EMD scenario, IMFs were extracted separately for FHR and UC series employing independent instances of EMD implementation; providing 80 features in total. Similar to BEMD case, classification performance of EMD was determined for both balanced and unbalanced data. It was observed that BEMD outperformed EMD for both balanced and unbalanced data for all classifiers. For example, 30% increase in sensitivity, 38% increase in specificity, 25% increase in AUC, and 37% decrease in MSE was observed for decision tree classifier for BEMD as compared to EMD-based features extraction (see section 6 of the Supplementary Material for more details). This was expected because BEMD ensures mode alignment both across FHR and UC channels as well as across different subjects. The EMD based method, on the other hand, would not achieve mode alignment across FHR and UC channels resulting in the sub-optimal performance. These results signify the importance of BEMD adaptation for an application as that of the current study.

## 7. Discussion

### 7.1. Main Findings

In the present study, we have employed multivariate extension of EMD algorithm to capture and utilize the complex dynamics of UC-FHR interactions for the purpose of improved classification of fetal states. Owing to the data-driven and fully multivariate nature of EMD, we have been able to decompose intrinsic oscillatory modes of raw FHR and UC data into multiple IMFs. More importantly, resulting IMFs have been demonstrated to exhibit “mode alignment,” which ensures similar frequency scales being aligned in same-indexed IMFs across two channels. That yields robust and physically meaningful time-frequency based features to be employed with machine learning classifiers for better classification of “vaginal vs. cesarean section” deliveries. Consistent with our hypothesis, we observed that empirically derived IMFs of distinct spectral characteristics embed unique features sensitive to the intrapartum dynamics. The present study adds another layer to the existing literature suggesting novel features of data-driven oscillatory components of CTG variabilities along with state-of-the-art machine learning classifiers to be employed in the current clinical obstetric practice for evaluation of fetal status. With a classification accuracy of 98%, the present study demonstrates that proposed features might be useful adjunct to conventional biochemical and biophysical measures of fetus to design a decision support system for fetal well-being.

### 7.2. Spectral Analysis of CTG Dynamics

After its introduction in 1960s, EFM was envisaged to assist diagnosis of fetus related complications such as cerebral palsy, neonatal seizures, and brain damage. Currently it is the most prevalent approach used in routine maternal care worldwide with approximately 85% live births (Sartwelle and Johnston, 2018). Traditionally, a visual scrutiny of continuous or intermittent FHR is practiced by obstetricians and gynecologists to evaluate the fetal well-being based on its morphological features, for example, accelerations, decelerations, baseline rate, overall impression contraction frequency, variability and determine risk. Though CTG was introduced with the expectation to prevent cerebral palsy and perinatal mortality, Cochrane Collaboration Review reported its merits only in terms of reduced risk of neonatal seizures with no evidence of added benefit for what it was anticipated (Alfirevic et al., 2017). Further to this, recent years have evidenced a significant increase in cesarean deliveries and instrumental vaginal births (Vintzileos et al., 1995; Melman et al., 2013), mainly attributed to the mis-diagnosis and mis-interpretation of CTG owing to its intrinsic subjective variability. With the aim to reduce the variability factor of interpretation, various intrapartum FHR interpretation systems have been suggested including 3-tier and 5-tier systems (Pruksanusak et al., 2017). It was concluded that 3-tier system is more appropriate than 5-tier system to be adopted in daily obstetrics practice (Pruksanusak et al., 2017).

Parallel to other clinical domains, FHR research has also sought a paradigm shift from the “subjective interpretation” to “objective tools” using computerized strategies and to develop automatised early-warning decision support systems. Along these lines, Cerutti et al. (1989) and other colleagues performed a series of studies employing spectral analysis of FHR to evaluate the clinical relevance of abdominal fetal ECGs during the antepartum period. They adopted auto-regressive model based spectral analysis, and analogous to adults, three significant patterns for fetal heart rate variability (HRV) spectrum were observed across VLF (< 0.03 Hz), LF (0.04–0.15 Hz), and HF (0.2–0.4 Hz) ranges. LF and HF powers were established to be sensitive to the sympathetic and parasympathetic activation, and that the LF/HF power ratio represents the sympathovagal balance in response to HRV. These studies also found that, additional to classical parameters e.g., mean and variance of RR intervals, the power spectral densities across these frequency ranges, and the LF/HF ratio index might be complementary to provide deeper understanding of autonomic nervous system of the fetus and its associated pathological factors.

Given that FHR patterns embed nonlinear dynamics (Choi and Hoh, 2015; Gonçalves et al., 2016), a study by Signorini et al. (2003) utilized an integrated approach of linear (autoregressive spectral power) and nonlinear [approximate entropy (ApEn)] models. The results evidence that examination of both signal variability and regularity seems to has potential to underline pathological conditions e.g., higher ApEn value represents increased irregularity, and lower LF power corresponds to the reduced contribution of the neural sympathetic drive under the pathological FHR. Various other nonlinear descriptors have also been used in literature to quantify FHR dynamics, including Lempel Ziv complexity (Spilka et al., 2012), multifractal approaches (Doret et al., 2011).

One fundamental limitation of spectral parameters is their derivation across arbitrary defined frequency bands which were actually adopted from adult HRV analysis and still remains controversial (Doret et al., 2015). As a matter of fact, few studies attempted multiscale analysis to estimate FHR dynamics over a wide range of scales. For example, variants of entropy based models [ApEn and Sample entropy (SamEn)] were found to be sensitive to short-term and long-term FHR patterns associated with normal and distressed fetuses of gestational ages of 30–35 weeks (Ferrario et al., 2006).

### 7.3. Machine Learning for CTG Analysis

It is not possible to make a direct comparison of the current study with studies of other researchers because of different methodologies and hypotheses. However, an indirect comparison in terms of the automatic characterization and classification of CTG based on machine learning tools to extract decision making features is outlined below.

An artificial neural network (ANN) was employed to differentiate normal conditions from pathological fetuses (Magenes et al., 2001), and nonstress tests (Kol et al., 1995). A sensitivity of 88.9% and a false-positive rate of 4.3% was achieved to discriminate between normal and abnormal nonstress test (Kol et al., 1995). In another study, Georgieva et al. (2013) also trained a feed-forward ANN to investigate FHR recordings and to predict adverse labor outcomes by extracting a set of six features. Later, these features were combined with six clinical parameters to form a larger set of 12 features, followed by a principal component analysis (PCA) for feature reduction. With 36% mis-classification rate for test data recordings, overall sensitivity of 60.3% and specificity of 67.5% was achieved in Georgieva et al. (2013). Georgoulas et al. (2006b) and Georgoulas et al. (2006a) also proposed a novel processing method for FHR analysis using PCA to define descriptive features, and a SVM based classifier to predict the risk of metabolic acidosis; best performance reported was 78% in terms of AUC. In a similar study, Ocak (2013) extracted features from FHR and UC signals, and performed classification using SVM followed by a feature reduction scheme (Xu et al., 2014) using genetic algorithm; achieving a high classification accuracy of 99.3% and 100%, respectively, for normal and pathological fetuses. Another study used particle swarm optimization and binary decision tree along with SVM to evaluate the fetal state (Yilmaz and Kilikçier, 2013) and achieved a classification accuracy of 91.62%.

With the aim to discriminate an abnormal pregnancy from the normal one, Spilka et al. (2014) adopted a random forest classifier along with the latent class analysis. Similarly, a sparse SVM classifier was adopted in a study for selection of most significant and relevant subset of features from a large number of linear, nonlinear and multifractal features for fetal acidosis detection (Spilka et al., 2017) and achieved a satisfactory accuracy (73% sensitivity and 75% specificity). In another study, Spilka et al. (2013) reported sensitivity and specificity of 64.1% and 65.2%, respectively, based on a combination of 50 classical morphological, frequency-domain and nonlinear features. Classification was performed using nearest mean classifier with AdaBoost. Similarly, another study by Spilka et al. (2012) reported a high classification accuracy of 73.4% sensitivity and 76.3% specificity by extracting a set of 33 conventional and nonlinear features. Stylios et al. (2016) extracted 54 discriminating features and observed that the application of only 3 features might provide sensitivity of 68.5% and specificity of 77.7%. With the aim to assess fetal hypoxia, an image-based time-frequency (IBTF) approach along with genetic algorithm was suggested by Cömert et al. (2018). It was reported that the classification of 15 IBTF features using a least square SVM classifier provided sensitivity of 63.45% and specificity of 65.88%.

With the objective to differentiate vaginal vs. cesarean delivery, recently Fergus et al. (2018) has suggested the use of an ensemble model comprising a combination of three classifiers applied to a set of features extracted from raw FHR tracings. Subsequently, a RFE scheme was followed to remove those features with low discriminating power. It was reported that the combination of Fisher linear discriminant analysis (FLDA), random forest (RF) and SVM achieved the best performance with sensitivity of 87%, specificity of 90%, AUC of 96% and MSE of 8%.

By far the majority of aforementioned studies focused on dynamic features of FHR traces in isolation, and did not incorporate responses of fetus to UCs. Analogous to adult HRV, FHR is also sensitive to external stimulus (Romano et al., 2006). Given the FHR deceleration follows a UC, the later provokes a transient hypoxia to fetus resulting in FHR variations; suggesting UC as a strong modulator of FHR (Romano et al., 2006). To this, the spectral estimation was performed using parametric and non-parametric time-varying approaches of short-time Fourier transform and autoregressive models across traditional frequency ranges (Romano et al., 2006). A significant increase in the FHR variability power was observed in “active” state as compared to the “quiet” state across LF and HF ranges (i.e., 0.03–0.2 and 0.2–1 Hz). Similar results were also found for distress fetuses (Warrick et al., 2009), with the application of a system identification approach estimating impulse response function, in terms of higher gain and prolonged delay between FHR and UC. The value of high gain corresponds to the strong relation, and a long delay represents the increased latency of FHR response to the UC. The authors also incorporated FHR baseline dynamics and the FHR variability along with the system identification approach to develop a larger set of features with the aim to classify normal vs. hypoxic fetuses. The application of SVM classifier detected half of the pathologies at least 1 h 40 min before the delivery. Despite having promising performance, these studies are limited by the fact that they mostly develop features across traditional frequency ranges. Further to that adopted methodologies assumed UC-FHR relations to be linear and stationary in nature, except few attempts which focused on the non-stationarity. For example, a BPRSA approach was adopted to assess the coupling of fetal ECG and uterine activity (Casati et al., 2014).

Though various studies have successfully achieved adequate accuracy, still there is a space for improvement, specifically for the delineation of vaginal vs. cesarean dynamics. Contrary to Fergus et al. (2018) which examined only FHR dynamics, the present study investigated nonlinear and nonstationary UC-FHR couplings using the multivariate extension of EMD. For this, scale-matched empirically derived modes of FHR and UC were examined to develop a set of unique features which have previously been applied to quantify various physiological systems (Saleem et al., 2016, 2018; Bhattacharyya and Pachori, 2017). We observed that AdaBoost classifier performs on-par with the best accuracy i.e., 91.8% sensitivity, 95.5% specificity, 98% AUC and 5% MSE.

### 7.4. Physiological Interpretation of IMFs and Their Associated Features

A recent trend in the spectral analysis of many biological and physiological systems has been to focus on the significant oscillatory modes rather than contiguous frequency ranges of the underlying dynamics. For example, recent work in cerebral hemodynamics have shown the superiority of utilizing oscillatory components extracted via principal dynamic modes analysis (Marmarelis et al., 2012; Saleem et al., 2017; Hameed et al., 2018; Shahzad et al., 2018), and Hilbert-Huang transformation based multimodal analysis (Novak et al., 2004). It is reported that control of various physiological mechanisms may be characterized by discrete oscillatory dynamics of the underlying time series rather than across a-priori defined traditional frequency bands e.g., sympathetic cerebrovascular control was associated with a low-pass and 0.03 Hz oscillatory components of the blood pressure control (Saleem et al., 2017). Similarly, 0.2 Hz oscillations of mechanical arm of the baroreflex loop were affected by orthostatic challenges (Shahzad et al., 2018).

Akin to these physiological systems, fetus dynamics also comprise biological oscillations e.g., labor contraction of uterus has been modeled as a 0.008 Hz oscillatory feedback system (Maeda, 2013). Along these lines, the present study also hypothesized that “vaginal vs. cesarean” dynamics might be differentiated by complex features of spectral oscillations of UC-FHR couplings. To achieve this, we adopted an EMD based approach, whose power mainly lies in its data-driven nature, to estimate inherent oscillatory components, namely IMFs. Each IMF carries inherent discrete scales present in input data, and its characteristics might be affected by certain physiological mechanisms. For example, IMFs of very-low-frequency (i.e., <0.03 Hz) might be influenced by thermoregulatory homeostasis (Gonçalves et al., 2013; Romano et al., 2016), and IMFs of low-frequency of 0.03–0.15 Hz might represent the controlling effect of neural sympathetic activity and vasomotor control (Signorini et al., 2003, 2014; Magenes et al., 2004; Cesarelli et al., 2010). Similarly, IMFs embedding 0.15–0.5 Hz oscillations may characterize mechanical attributes of maternal breathing and fetal movements (Signorini et al., 2003, 2014; Magenes et al., 2004; Cesarelli et al., 2010), whereas high-frequency IMFs of 0.5–1 Hz might be affected by the fetal respiration related vagal activity (Signorini et al., 2003, 2014; Magenes et al., 2004; Cesarelli et al., 2010). Future studies might target to quantify the precise biological origins of these IMFs, for example, by enhancing certain frequency components of UC-FHR couplings. One approach to this direction might be to apply external stimuli to trigger a specific physiological mechanism to observe the corresponding changes in IMF features (Magenes et al., 2004).

Though FHR and UC time series can embed many IMFs, the present study noticed that important data scales, which could help examine fetal dynamics for “vaginal vs. cesarean” classification, mostly resided in first few IMFs containing high frequency modes. However, this does not mean that remaining IMFs are of no biological or physiological relevance, instead it could imply that information in those IMFs does not contribute much toward classification task at hand.

The precise physiological interpretation of adopted features of IMFs is imperceptible, however plausible explanation is as follows. Larger MJiA values of higher indexed IMFs might represent that low-frequency activities of both FHR and UC are mutually coherenced. Positive MmAM values may indicate the highly consistent variations in amplitudinal characteristics of both FHR and UC, whereas the negative MmAM values represent that these amplitudinal fluctuations might be in opposite directions. Negative MmAM values across the cesarean cohort shows that variations in signal strengths of FHR and UC are less synchronized as compared to that of the vaginal group. VmAM might represent the deviation of signal strengths. Larger VmAM values across low-indexed IMFs show that amplitudinal variations of high frequency FHR and UC oscillations are highly heterogeneous. WMF represents the strengths of synchronization between amplitude and frequency characteristics of a specific spectral oscillation. In FHR, both amplitude and frequency were highly coherenced across low- and high-frequency oscillations as compared to that across middle-frequency oscillations. For UC dynamics, low-frequency oscillations demonstrated greater amplitude-frequency coherence in the vaginal group. Similar to FHR, UC also demonstrated reduced amplitude-frequency synchronization across middle-frequency oscillations. ME represents the relative distribution of power across different frequency components. Interestingly, high-indexed IMFs of both FHR and UC carry most of the power, indicating that low-frequency control mechanisms contribute strongly to both FHR and UC dynamics. PSI represents the phase-synchronization between same-indexed IMFs of FHR and UC. Greater PSI across high-indexed IMFs shows that low-frequency oscillations of FHR and UC are highly synchronized.

### 7.5. Complexity Analysis of the Proposed Scheme

Since findings of the present study are quite encouraging suggesting the application of the proposed scheme in ongoing CTG research, it is worth-mentioning its real-time implementation and computational cost. EMD-based algorithms, especially their real-time implementations, are inherently computationally very expensive. Moreover, owing to their completely data-driven nature, computational requirements of EMD-based methods might be hard to establish explicitly since they vary with the complexity (number of oscillations) of the input signal. Though, the EMD and its multivariate extensions, in their original formulation were designed for batch processing, FPGA based architecture for online and real-time computation of the BEMD has recently been proposed (Malik et al., 2019). It would make sense to use that architecture in BEMD applications requiring online and real-time processing, including the application addressed in the present work. That would be a good avenue for future research.

It is also relevant to mention that the BEMD operation, which is exclusively used for signal decomposition, is not dependent on the length of the input time series, rather it depends on the number of extrema in the input signal. Note that the BEMD sifting operation stops when the residual signal is devoid of any rotations (oscillations in 2D). For classification task, according to the statistical heuristics, data available to train machine learning classifiers should contains ample number of training instances in comparison to the number of features. For example, a rule-of-thumb is that data samples should be at least 10 times more than the number of features (Hua et al., 2004). In the current case of 40 features, minimum 400 data samples would be sufficient for appropriate training of the machine learning classifiers. Our classification experiments with cross-validation on unseen data demonstrated the sufficiency of the training data for the classification task at hand. In addition, previous studies (Fergus et al., 2018) also provided evidence of adequacy to train classifiers using the same data for the similar classification task.

Regarding the computational cost of the BEMD decomposition, a detailed description is provided in ur Rehman et al. (2014). Briefly, the numerical complexity of BEMD for the input signal of *T* samples, number of projections *V*, total number of *M* IMFs, and the number of detected extrema *M*_*k*_(*d*_*m, k*_, *v*) in the *v*th projection of the *m*th IMF and the *k*th iteration is given by,

(15) $$\begin{array}{l} C=\overset{M}{\underset{m=1}{\sum }}\overset{K_{m}}{\underset{k=1}{\sum }}T\left(11V+2\right)+\overset{M}{\underset{m=1}{\sum }}\overset{K_{m}}{\underset{k=1}{\sum }}\overset{V}{\underset{v=1}{\sum }}15M_{k}\left(d_{m,k},v\right). \end{array}$$

Note that the complexity of BEMD depends on the number of detected extrema of the input signal which makes it hard to predict or specify the complexity of the method for an unseen class of signals.

The average training time for a machine learning classifier for a 40 dimensional feature matrix containing 404 training samples was found to be approximately 121 ms. Whereas, the computational time at the test stage for 102 test instances was approximately 11 ms on a machine with 2.30GHz i5-6200U CPU and 8GB RAM running MATLAB (version R2018a; Mathworks). Of note, the difference between training and test time increases proportionally for all machine learning classifiers when the number of training and test instances are increased. As training is performed in an off-line manner, the real-time performance of these classifiers depends upon the computational time requirements at the test stage. Mostly, the C/C++ versions of these classification methods give real-time performance at the test stage for a large number of test instances, thus, favoring their application in on-line prediction systems (Lu et al., 2010).

### 7.6. Conclusion

To conclude, the adaptive decomposition of FHR and UC interactions provides basis functions, in terms of IMFs, which might be used to derive robust features for classification of vaginal vs. cesarean delivery dynamics. The results of the present study signify that appropriate machine learning and signal processing algorithms might help to understand CTG variability, and to improve inter- and intra-observer agreement for the CTG interpretation.

## Data Availability

Data examined in this study is freely available and can be downloaded from Physionet: http://www.physionet.org/physiobank/database/ctu-uhb-ctgdb/

## Ethics Statement

The data collection procedures were approved by the Institutional Board of the University Hospital in Brno, Czech Republic. A written informed consent was obtained from all women participants. Data were collected at the obstetrics ward of the University Hospital in Brno (UHB) in collaboration with the Czech Technical University (CTU), Prague between April 2010 and August 2012. All CTG recordings were evaluated by 9 senior obstetricians adhering to FIGO guidelines practiced in Czech Republic. An anonymous unique identifier was used to match intrapartum CTG waveforms to the clinical data by the hospital information system.

## Author Contributions

SS, SN, AS, NuR, and JM: contributed to the study design; SS, SN, TM, AS, NuR, and JM: contributed to the data analysis; SS, SN, TM, AS, NuR, and JM: contributed to the data interpretation; SS, SN, TM, AS, NuR, and JM: contributed to the manuscript drafting. All authors gave the final approval for publication.

### Conflict of Interest Statement

The authors declare that the research was conducted in the absence of any commercial or financial relationships that could be construed as a potential conflict of interest.
