# Supplementary material for: A Strategy for Classification of “Vaginal vs. Cesarean Section” Delivery: Bivariate Empirical Mode Decomposition of Cardiotocographic Recordings
Source: Front Physiol. 2019 Mar 19;10:246. doi: 10.3389/fphys.2019.00246 (PMC6433745; doi:10.3389/fphys.2019.00246)
Supplement: Supplementary file 1 [file Data_Sheet_1.PDF]

Supplementary Material

1 RECURSIVE FEATURE ELIMINATION (RFE)

The detailed procedure of support vector machine (SVM) based RFE (43) is presented below.

Input: Let $[\mathbf{f}_1\ \ \mathbf{f}_2 \cdots \mathbf{f}_k]^T \epsilon\ \ F$ be the training feature set, and $[y_1\ \ y_2 \cdots y_k]^T \epsilon\ \ Y$ be the corresponding labels.

Output: Reduced feature set R.

Algorithm 1 RFE

1: Update F with only the subset of surviving features: $F = F - S$,
2: learn the classifier $\alpha = fit_{SVM(F,Y)}$,
3: Compute the weight vector $w = \sum_k \alpha_k y_k \mathbf{f}_k$, and the ranking criteria $c_i = (w_i)^2$,
4: identify the feature with the smallest ranking criterion $f_i = \text{argmin}(c_i)$ and delete the feature with the lowest ranking criterion from S,
5: repeat steps 1–4 until the minimum number of features in S are achieved.

2 BELSLEY COLLINEARITY TEST

To test the collinearity of selected features, we employed the collinearity test by Belsley et al. (44), known as the Belsley collinearity diagnostics. Given the design matrix X (of features), the Belsely collinearity diagnostics detects the sources of collinearity and provides a measure of their strength. This test uses the condition indices to identify the number and strength of the relationships between the variables. To detect the interdependent variates and, to estimate the level of degradation in regression due to the dependencies, variance decompostion propotions are required.

Let $S_{(1)} \geq S_{(1)} \geq ...S_{(n)}$ be singular values for X with n columns, then the condition indices for the variables are $S_{(1)}/S_{(j)},\ j = 1, ..., n$. All condition indices are in the range of 1 and $S_{(1)}/S_{(n)}$. The singular value decomposition of the scaled desgin matrix X yeilds the variance decomposition propotions matrix V, which comprises of orthonormal eigenvectors of $X^{'}X$.

We employed the Belsley collinearity diagnostics (44) on the data from all subjects using all extracted features. Figure S1 presents the condition indexes for the scaled matrix X, where a red line shows the threshold for the condition index based on the ranges reported by Belsley et al. (44). Only a handful of dependencies were observed in the design matrix X.

To probe further and investigate interdependent attributes, the variance decomposition proportions are computed and visualized in Figure S2. It can be observed from Figure S2 that first few features exhibit near dependencies. These attribute pairs can be localized by thresholding the variance decomposition proportions with a threshold of 0.5, as discussed in (44).

3 SYNTHETIC MINORITY OVER-SAMPLING (SMOTE)

The procedure for generating synthetic samples from SMOTE (45) is presented below,

Input: the number of minority class samples T, the number of nearest neighbors k and the number of SMOTE samples to be generated N.

Output: a set of synthetic generated samples M.

Algorithm 2 SMOTE

for $i \leftarrow 1$ to T,
compute k nearest neighbors for i,
while $N \neq 0$,
$M \leftarrow \mathbf{f} + r(\mathbf{f} - \mathbf{f}_{\text{nn}})$,
$N = N$-1,
end **while**
end **for**

where **f** represents the feature vector under consideration, and $\mathbf{f}_{\text{nn}}$ represents the feature in the nearest neighbor.

4 FOURIER SPECTRUM OF INTRINSIC MODE FUNCTIONS

Figure S3 represents the Fourier spectrum of five IMFs for 10 different subjects. All five IMFs were found having distinct spectral peaks. It is evident that IMFs were scale-matched across different subjects.

5 SELECTION OF NUMBER OF INTRINSIC MODE FUNCTIONS (M)

Figure S4 shows the trend of AUC values for a decision tree classifier for various selections of M (i.e., number of IMFs). Highest AUC (78%) was observed for M=5 and smallest AUC (31%) was found for M=1. This observation shows that relevant important information needed for good classification performance is present in the first five IMFs, and therefore led to the selection of M=5 in this study.

6 CLASSIFICATION PERFORMANCE OF EMD- VS. BEMD-BASED FEATURES

Figure S5 shows the performance of machine learning classifiers based on EMD-based features for both balanced and unbalanced datasets. It was observed that classification accuracy was higher for BEMD-based features across all classifiers for both balanced and unbalanced datasets (Figure S6).

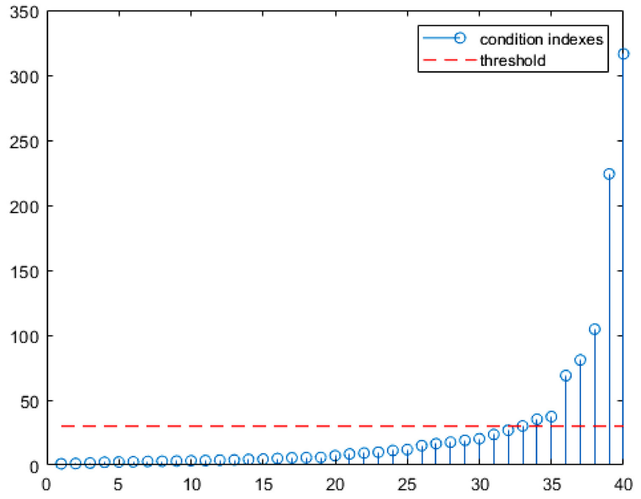

Figure S1. Condition indexes for features..

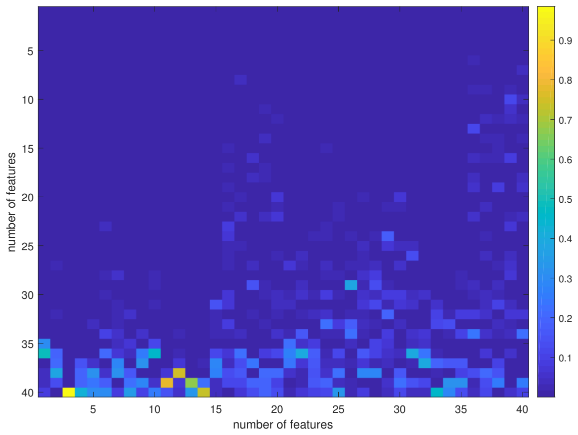

Figure S2. Variance decomposition proportions for the scaled matrix X. Higher values of variance decomposition proportions are shown in darker shades of yellow..

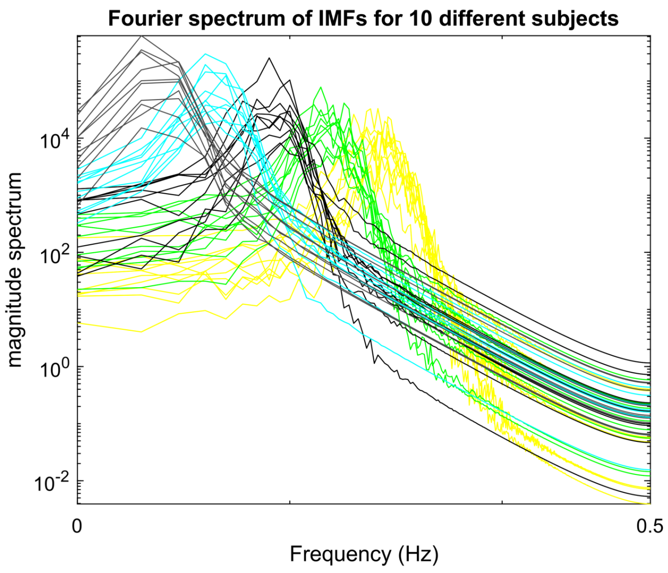

Figure S3. Fourier spectrum of five IMFs for 10 different subjects.

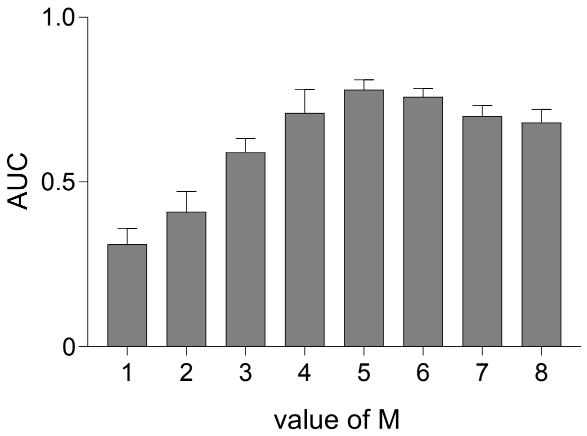

Figure S4. AUC as a function of M for balanced dataset for decision tree classifier. AUC, area under the ROC curve.

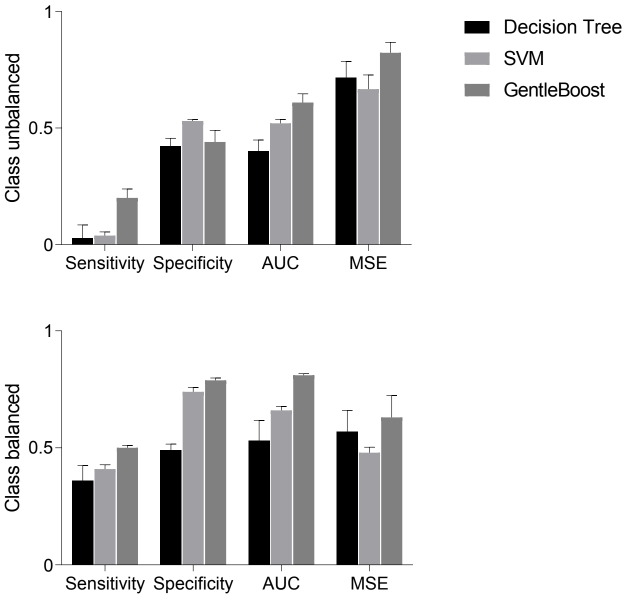

Figure S5. Classification performance based on features extracted from EMD.

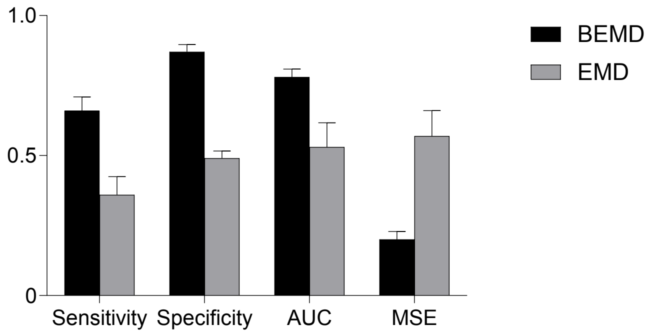

Figure S6. Classification performance comparison between BEMD- and EMD-based features for balanced data for decision tree classifier.
